# Mefunidone Attenuates Tubulointerstitial Fibrosis in a Rat Model of Unilateral Ureteral Obstruction

**DOI:** 10.1371/journal.pone.0129283

**Published:** 2015-06-04

**Authors:** Chunyan Liu, Wenjuan Mei, Juan Tang, Qiongjing Yuan, Ling Huang, Miaomiao Lu, Lin Wu, Zhangzhe Peng, Jie Meng, Huixiang Yang, Hong Shen, Ben Lv, Gaoyun Hu, Lijian Tao

**Affiliations:** 1 Department of Nephrology, Xiangya Hospital, Central South University, Changsha, Hunan, China; 2 Department of Pathology, Hunan University of Chinese Medicine, Changsha, Hunan, China; 3 Department of Respiration, Xiangya Hospital, Central South University, Changsha, Hunan, China; 4 Department of Gastroenterology, Xiangya Hospital, Central South University, Changsha, Hunan, China; 5 Institute of Medical Sciences, Xiangya Hospital, Central South University, Changsha, Hunan, China; 6 Department of Hematology, The Third Xiangya Hospital, Central South University, Changsha, Hunan, China; 7 Department of Medical Chemistry, School of Pharmaceutical Sciences, Central South University, Changsha, Hunan, China; 8 State Key Laboratory of Medical Genetics of China, Central South University, Changsha, Hunan, China; National Institute of Health and Medical Research, FRANCE

## Abstract

**Background:**

Inflammation has a crucial role in renal interstitial fibrosis, which is the common pathway of chronic kidney diseases. Mefunidone (MFD) is a new compound which could effectively inhibit the proliferation of renal fibroblasts *in vitro*. However, the overall effect of Mefunidone in renal fibrosis remains unknown.

**Methods:**

Sprague-Dawley rats were randomly divided intro 6 groups: sham operation, unilateral ureteral obstruction (UUO), UUO/Mefunidone (25, 50, 100mg/kg/day) and UUO/PFD (500mg/kg/day). The rats were sacrificed respectively on days 3, 7, and 14 after the operation. Tubulointerstitial injury index, interstitial collagen deposition, expression of fibronectin (FN), α-smooth muscle actin (α-SMA), type I and III collagen and the number of CD3+ and CD68+ cells were determined. The expressions of proinflammatory cytokines, p-ERK, p-IκB, and p-STAT3 were measured in human renal proximal tubular epithelial cells of HK-2 or macrophages.

**Results:**

Mefunidone treatment significantly attenuated tubulointerstitial injury, interstitial collagen deposition, expression of FN, α-SMA, type I and III collagen in the obstructive kidneys, which correlated with significantly reduced the number of T cells and macrophages in the obstructive kidneys. Mechanistically, Mefunidone significantly inhibited tumor necrosis factor-α (TNF-α-) or lipopolysaccharide (LPS)-induced production of proinflammatory cytokines. This effect is possibly due to the inhibition of phosphorylation of ERK, IκB, and STAT3.

**Conclusion:**

Mefunidone treatment attenuated tubulointerstitial fibrosis in a rat model of UUO, at least in part, through inhibition of inflammation.

## Introduction

The incidence of chronic kidney disease (CKD), which can lead to end-stage renal disease (ESRD), has increased in recent years and has become a worldwide public health problem [1, 2]. Renal fibrosis is the ultimate feature of CKD and the common cause of renal failure. Compared to glomerulosclerosis, interstitial fibrosis is closely related to ESRD. The main pathological features of renal interstitial fibrosis include infiltration of inflammatory cells, excessive accumulation of extracellular matrix (ECM), tubular atrophy, and proliferation of fibroblasts. Although ESRD cannot be reversed, renal interstitial fibrosis may be reversible [3].

Previous studies established that anti-fibrotic drugs can interfere with several aspects of the pathogenesis of renal fibrosis. These include anti-inflammatory agents, antagonists of transforming growth factor-beta (TGF-β) action, inhibitors of tyrosine kinase growth factor receptors, intracellular signaling pathways inhibitors, agents degrading extracellular matrix, inhibition of collagen receptors, and so on [4]. But some studies showed that pharmacological treatment when aiming at one molecule at a time was probably not always the good strategy for treatment [5]. The role of multi-target compounds is more anticipated than single-target compounds. Thus, effective treatment of renal fibrosis requires multi-channel drugs.

In recent years, two multi-channel anti-fibrosis drugs of pirfenidone (PFD) and fluorofenidone (AKF-PD) have been developed. Pirfenidone (PFD) is recognized as the most promising anti-fibrotic drug in clinical application [6]. PFD had been approved for the treatment of idiopathic pulmonary fibrosis (IPF) [7]. Further, PFD was proved to be effective in patients with focal segmental glomerulosclerosis [8] or diabetic nephropathy [9]. AKF-PD (fluorofenidone), a novel synthetic compound, was screened by our group. Our works showed that AKF-PD had similar anti-fibrotic effect as PFD [10–16]. However, the effective dose of PFD and AKF-PD in the rat model of UUO was as high as 500mg/kg/day [10, 17]. Therefore, it has very important significance to development novel anti-fibrosis drugs with lower effective dose but with the same or even higher therapeutic efficacy.

Based on AKF-PD, we improved the chemical structures and prepared a series of compounds with higher water solubility which may have higher activities *in vivo*. Among the compounds tested, Mefunidone [1-(4-((3-(4-methylpiperazin-1-yl)propyl)amino)benzyl)-5-(trifluoromethyl)pyridin-2(1*H*)-one] showed the higher inhibitory activity against renal fibroblasts proliferation, the IC_50_ of which was 0.34mM while the IC_50_ of PFD or AKF-PD was 2.92mM or 2.64mM respectively. Notably, the acute virulent test and the long-period virulent test of Mefunidone showed a lower toxicity as compared to PFD or AKF-PD (data not shown). However, the treatment effects of Mefunidone as a novel anti-fibrotic on tubulointerstitial fibrosis need to be further identified.

UUO is a well-characterized animal model of renal tubulointerstitial fibrosis [18]. So we wanted to establish UUO model to investigate the effect and molecular mechanism of Mefunidone on interstitial fibrosis. Tubulointerstitial fibrosis is typically initiated by the infiltration of inflammatory cells. Early studies established that inflammatory response occurs throughout the development of CKD. Infiltrated inflammatory cell plays an important role in chronic inflammation and fibrosis in the kidney diseases [19]. Mechanistically, proinflammatory mediators and growth factors produced by inflammatory cells not only promote the proliferation of renal fibroblasts and the transdifferentiation of renal tubular epithelial cells, but also stimulate local cells to produce transforming growth factor (TGF)-β and other fibrogenic factors [20]. In addition, activated renal tubular epithelial cells also produce inflammatory factors, such as IL-1, TNF-α, TGF-β1, and MCP-1 [21]. Accordingly, many anti-inflammatory compounds are effective in the treatment of CKD [22]. Janus kinase-signal transduction and transcription activator (JAK-STAT), mitogen-activated protein kinases (MAPKs), and nuclear factor-κB (NF-κB) are three important intracellular signaling pathways that play pivotal roles in the regulation of inflammation [23, 24]. The aim of present study was to investigate whether Mefunidone could attenuate tubulointerstitial fibrosis and associated inflammation in a rat model of UUO.

## Materials and Methods

### Animals and experimental protocol

A total of 90 male Sprague-Dawley rats (8 weeks old, 180–200g) were purchased from Shanghai SLACCAS Laboratory Animal Co., Ltd. (Shanghai, China). As described previously [25], 75 rats underwent UUO by ligation of the left ureter. Sham-operated rats (n = 15) underwent all procedures except ureteral ligation, and were used as controls. After the operation, UUO rats were randomly divided into five groups: UUO with drug solvent treatment (n = 15), UUO treated with Mefunidone (25, 50, and 100 mg/kg/day (n = 15, respectively), Lot No. 20121222, synthesized by School of Pharmaceutical Sciences, Central South University, China), and UUO treated with PFD (n = 15, 500 mg/kg/day, Lot No. 20080402, synthesized by School of Pharmaceutical Sciences, Central South University). Mefunidone was prepared as hydrochloride solution and diluted with the vehicle solvent (1% carboxymethyl cellulose sodium; CMC-Na). PFD was dissolved in the vehicle solvent (1% CMC-Na). All drugs and vehicle treatment (1% CMC-Na) groups were administered by oral gavage once daily from the first day after the operation. The rats were sacrificed respectively on days 3, 7, and 14 after the operation (n = 5 at every time point). The obstructed kidney was cut along the coronal plane and half of the kidney was fixed in 10% neutral buffered formalin for paraffin embedding. The remaining renal cortex was snap-frozen in liquid nitrogen for further research. The rats were maintained under 12h light/dark cycles with food and water *ad libitum*. All animal procedures and experimental protocols complied with NIH guidelines. All studies were approved by the Institutional Animal Care and Use Committee of Xiangya School of Medicine, Central South University.

### Renal pathology and immunohistochemistry staining

For pathological analysis, 4μm-thick renal sections were cut from the paraffin blocks. The HE-stained sections were evaluated semi-quantitatively, as described previously, to assess renal tubulointerstitial injury [26]. To further assess the degree of tubulointerstitial collagen deposition, Masson’s trichrome staining sections were graded as mentioned before [27]. To confirm the effect of Mefunidone on ECM deposition after obstructive injury, we used Sirius red polarization based on the procedure as described [28, 29].

DAB immunohistochemistry staining was processed using DAKO EnVision System (Dako Diagnostics, Zug, Switzerland) for renal immunohistochemical assay. The slides were incubated with a primary antibody against collagen I (1:500; Abcam, Cambridge, UK), collagen III (1:800; Abcam), CD3 (1:100; BD Pharmingen, CA), or CD68 (1:200; AbD Serotec, Kidlington, UK). Given the homogenicity of the target proteins’ staining, the interstitial staining of collagen I and collagen III was measured as described by a blinded observer using computerized morphometry (Image Pro-Plus 6.0 software, Media Cybernetics, Bethesda, MD) [30]. The interstitial staining cell numbers of CD3 and CD68 in 20 randomly selected fields at 400× magnifications in the cortex and outer medulla were analyzed by a blinded renal pathologist and nephrologist as described [31].

### Cell cultures

NRK-49F cells ((a rat kidney fibroblast cell line, ATCC, Rockville, MD) were cultured in Dulbecco's Modified Eagle's Medium (DMEM; Gibco BRL, Gaithersburg, MD) supplemented with 5% fetal bovine serum (FBS; Gibco BRL), 100U/ml penicillin, and 100μg/ml streptomycin. HK-2 cells (ATCC) were grown in DMEM/F12 (Gibco BRL) supplemented with 10% FBS, 100U/ml penicillin (Gibco BRL), and 100U/ml streptomycin (Gibco BRL). Primary peritoneal macrophages were isolated from BALB/C mice purchased from Shanghai SLACCAS Laboratory Animal Co., Ltd. as previously described [32]. Mouse macrophages were pre-cultured in RPMI 1640 medium (Gibco BRL) supplemented with 10% FBS (Gibco BRL) and 2mM glutamine.

### Cell proliferation assay and cytotoxicity assay

The in vitro proliferation of NRK-49F treated with Mefunidone and PFD was assessed by Cell Proliferation ELISA, BrdU (chemiluminescence) kit (Cat.No.11669915001; Roche, Basel, Switzerland) following manufacturer’s instructions. Light emissions of the samples were measured in a microplate luminometer (Synergy 2 Multi-Mode Microplate Reader; BioTek, Highland Park, TX) by BrdU incorporation following manufacturer’s instructions. The effect of Mefunidone and PDF on cell death of NRK-49F was assessed by Lactate Dehydrogenase (LDH) Cytotoxicity Assay Kit (Cat.No.C0016; Beyotime Biotechnology, Jiangsu, China) according to the manufacturer's instructions. The absorbance was measured at 490 nm by the microplate reader within 1 h. Experiments were performed in triplicate in three independent experiments.

### ELISA for cytokines

HK-2 cells were pretreated with Mefunidone at different concentrations (0.06, 0.12, and 0.18mM) and DXM (10^-6^M; Sigma-Aldrich, St.Louis, MO) was used as a positive control for 1h and then stimulated with 10ng/mL of recombinant human TNF-α (Gibco) for 24h. Supernatants were collected and stored at −80°C until used for cytokine determination. MCP-1 was determined by ELISA according to the manufacturer’s instructions (R&D Systems, Minneapolis, MN).

Mouse primary peritoneal macrophages were pretreated with Mefunidone (0.18mM) and DXM (10^-6^M) for 1h and then stimulated with 250ng/mL of LPS (from *Escherichia coli* serotype O111:B4; Sigma-Aldrich) for 24h. Supernatants were collected and IL-6 and TNF-α were determined using an ELISA kit (Invitrogen, Grand Island, NY).

### Western blot analysis

NRK-49F cells were pretreated with Mefunidone (0.18mM) or PFD (2mM) for 1h and then stimulated with 10ng/ml of recombinant human TGF-β1 for 48h. HK-2 cells were pretreated with Mefunidone at different concentrations (0.06, 0.12, and 0.18mM) for 24h or inhibitors for 1–2h and then stimulated with 10ng/ml of recombinant human TNF-α for 15, 30, or 60min. Primary peritoneal macrophages were pretreated with Mefunidone (0.18mM) and different inhibitors for 1–2h and then stimulated with 250ng/ml of LPS for 15, 30, or 60min.

Whole proteins from kidneys or cells were collected as described previously [33]. Membranes were incubated overnight at 4°C with the following specific primary antibodies against α-SMA (1:2,500; Sigma), fibronectin (1:400; Santa Cruz Biotechnology, Dallas, TX), phosphor-ERK1/2 (1:1,000; Cell Signaling Technology, Danvers, MA), ERK1/2 (1:1,500; CST), phosphor-IκB (1:1,000; CST), IκB (1:1,000; CST), phosphor-STAT3 (Ser727, 1:1000; CST), STAT3 (1:1000; CST) or β-tublin (1:1,000; Santa Cruz).

### RNA extraction and real-time PCR quantitation

Total RNA was isolated from kidney tissue using Trizol Reagent according to the manufacturer’s instructions (Invitrogen). For quantitative real-time PCR analysis, complimentary DNA (cDNA) was synthesized from 1ug of total RNA using a ReverseAid first strand cDNA synthesis kit (Thermo Scientific Fermentas; Thermo Fisher Scientific Inc., Waltham, MA) and analyzed using an SYBR Green PCR reagent kit (SYBR PremixEx Taq II, Takara, Japan) in a CFX96 Real-Time System (BIO-RAD Laboratories, Hercules, CA). The specific primers (collagen I: 5ʹ-TCAGGGGCGAAGGCAACAGT-3ʹ and 5ʹ-TTGGGATGGAGGGAGTTTACACGA-3ʹ; collagen III: 5ʹ-AAGGGCAGGGAACAACTGAT-3ʹ and 5ʹ-GTGAAGCAGGGTGAGAAGAAAC-3ʹ; β-actin: 5ʹ-GGCCAACCGTGAAAAGATGA-3ʹ, and 5ʹ-GACCAGAGGCATACAGGGACAA-3ʹ) were designed from the GenBank sequences and synthesized by Bio Basic (Generay Biotechnology, Shanghai, China). The quantity of mRNA was calculated based on the cycle threshold (CT) values which were standardized with the amount of the housekeeping gene β-actin. ΔCT was the value from the corresponding target gene’s CT value subtracting the CT value of the β-actin. Further calculation was performed using the 2^-ΔΔCT^ method, and the results were expressed as the n-fold difference relative to normal controls.

### Transfection and luciferase assay

Reporter plasmid pNF-κB-Luc (luciferase) was generously provided by the Department of Pathophysiology, Xiangya School of Medicine (Central South University, Changsha, Hunan, China). pRL-SV40 (Promega, Madison, WI) was used as an internal control. Lipofectamine Transfection Reagents (Invitrogen) were used. After transfection as previously described [34], cells were cultured overnight and treated with 10ng/ml TNF-α in the presence or absence of Mefunidone (0.06, 0.12, and 0.18mM) or DXM (10^-6^M) as positive control. Luciferase activity was assessed using a luciferase assay substrate (Promega) according to manufacturer’s instructions.

### Statistical analysis

Data were reported as means±standard deviation. Statistical analysis of data was performed with SPSS 18.0 software (SPSS Inc., Ill., USA). Comparison among groups was made with one-way ANOVA. Multiple-comparison tests were applied only when a significant difference was determined by the ANOVA; p<0.05 was considered significant. The cell experiment was repeated at least three times with similar results.

## Results

### Mefunidone did not affect the renal function in rats with UUO 14 days

We observed that UUO rats on day 14 had increased serum creatinine, which was in accordance with the previous studies [15, 35], but with no significant change in urea nitrogen compared to sham-operated rats. There were no significant differences in serum creatinine and urea nitrogen in Mefunidone- and PFD-treated groups compared to untreated UUO rats, suggesting that Mefunidone and PFD have no significant effect on the contralateral glomerular filtration rate (S1 Fig).

### Mefunidone attenuated tubulointerstitial fibrosis in UUO

**Fig 1:**
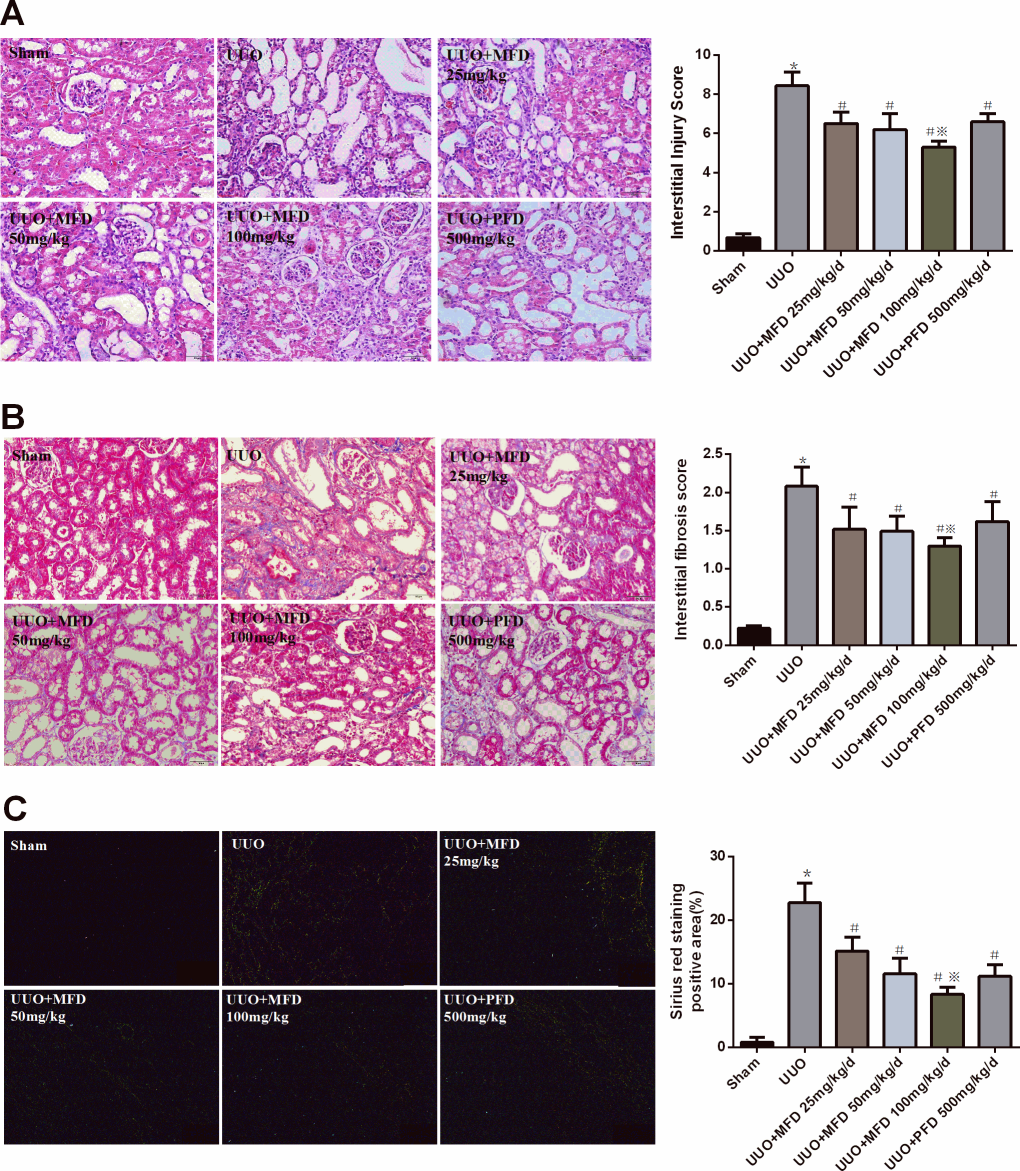
Mefunidone attenuated tubulointerstitial injury and extracellular matrix (ECM) deposition in the obstructive kidneys after UUO at 14 days. ***A*.** Mefunidone attenuated tubulointerstitial injury in the obstructive kidneys (HE staining of rats’ kidney sections, ×200). The column section indicated the interstitial injury score of the obstructed kidneys in each group. ***B*.** Mefunidone attenuated the deposition of ECM in the obstructive kidneys (Masson staining of rats’ kidney sections, ×200). The column section indicated the interstitial fibrosis score of the obstructed kidneys in each group. ***C*.** Mefunidone attenuated the deposition of ECM in the obstructive kidneys (Sirius red staining under polarized light; in green, yellow, or orange light, ×200). The column section indicated the Sirius red staining positive area of the obstructed kidneys in each group. All data were presented as means±SD, n = 5. (*p<0.05 compared to the sham group; #p<0.05 compared to the UUO group; ※p<0.05 compared to the PFD group.)

To assess the effect of Mefunidone on renal fibrosis *in vivo*, we treated UUO rats with different doses of Mefunidone. Between the rats in UUO group and the rats in Mefunidone or PFD group, there were no significant differences in body weight, gastrointestinal tract reaction and mental state (data not shown). The kidneys from UUO rats, but not sham-operated rats, developed tubulointerstitial injury, which is manifestated by tubular dilatation, tubular atrophy, tubular epithelial cell vacuolization, interstitial inflammatory cells infiltration, and interstitial fibrosis. Notably, Mefunidone 25, 50 and 100mg/kg/day treatment significantly attenuated tubulointerstitial injury at 14 days after UUO, as compared to vehicle solvent. The PFD treatments also attenuated the renal pathological alterations and reduced the tubulointerstitial injury scores on UUO at 14 days. However, the tubulointerstitial injury score in the Mefunidone 100mg/kg/day group was significantly lower than that in the PFD group (Fig 1A).

To assess ECM deposition in the obstructed kidney, we used Masson’s trichrome staining and Sirius red polarization. Notably, the administration of Mefunidone (25, 50, and 100mg/kg/day) markedly decreased the score of interstitial collagen deposition and Sirius red staining positive area compared with the UUO group with vehicle solvent treatment. PFD treatment also significantly reduced ECM deposition (Fig 1B and 1C). However, the collagen deposition score and Sirius red staining positive area in the Mefunidone 100mg/kg/day group was significantly lower compared with the PFD group (Fig 1B and 1C).

**Fig 2:**
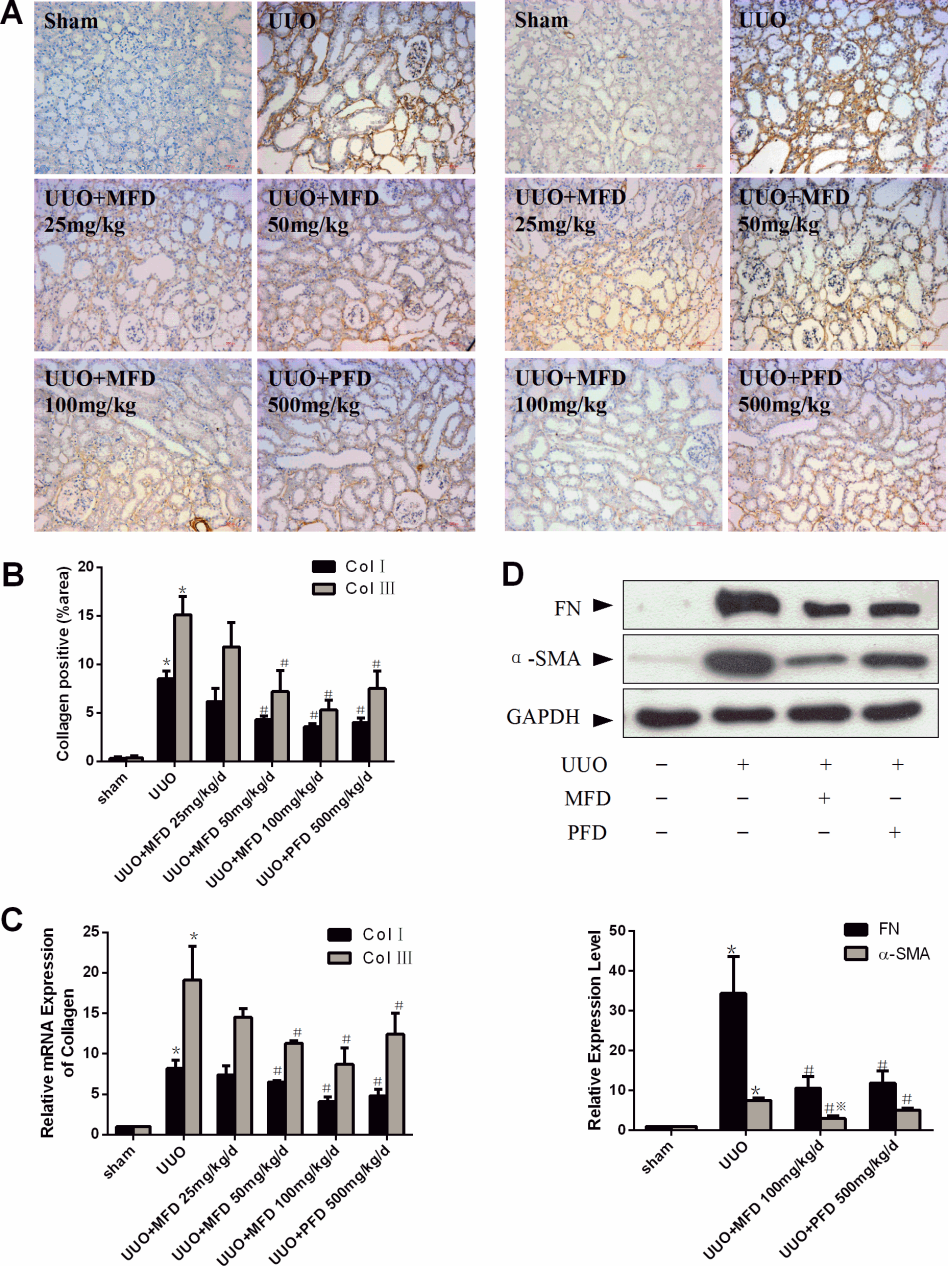
Mefunidone decreased collagen I, collagen III, α-smooth muscle actin (α-SMA) and fibronectin (FN) expression in the obstructive kidneys after UUO at 14 days. ***A*.** Mefunidone affected collagen I (left) and collagen III (right) protein expression in the obstructed kidneys stained by immunohistochemistry (×200). ***B*.** The histogram was the semi-quantitative analysis result of collagen I and collagen III protein expression in the kidneys. ***C*.** The histogram was the relative mRNA expression of collagen I and collagen III in the kidneys. ***D*.** Mefunidone decreased the protein expressions of α-SMA and FN in the obstructed kidneys as shown by Western blot. The column section indicated the relative expression level of the obstructive kidneys in each group. All data were presented as means±SD, n = 5. (*p<0.05 compared to the sham group; #p<0.05 compared to the UUO group.)

Collagen I and III are the major ECM components which accumulate in the kidney during renal tubulointerstitial fibrosis. Next, we assessed the protein expression of collagen I and collagen III by immunohistochemistry staining, and mRNA expression of collagen I and collagen III using real-time PCR 14 days after UUO. Medium and high doses of Mefunidone and PFD resulted in a significant reduction of interstitial collagen I and collagen III expression (Fig 2A, 2B and 2C). Together, immunostaining and real-time PCR revealed that Mefunidone treatment inhibited interstitial collagen I and collagen III expression.

Fibroblasts in the renal interstitium are considered as the principal source of fibrillar matrix (collagen types I and III). FN and α-SMA are two hallmarks of fibroblast activation [36]. Thus we next examined the protein expression of FN and α-SMA in the obstructive kidneys. We observed that both Mefunidone and PFD significantly reduced the protein expression of FN and α-SMA in the obstructive kidneys. In this context, Mefunidone has stronger inhibitory effect in the expression of α-SMA, as compared to PFD (Fig 2D).

### Mefunidone inhibited renal fibroblast proliferation and activation

**Fig 3:**
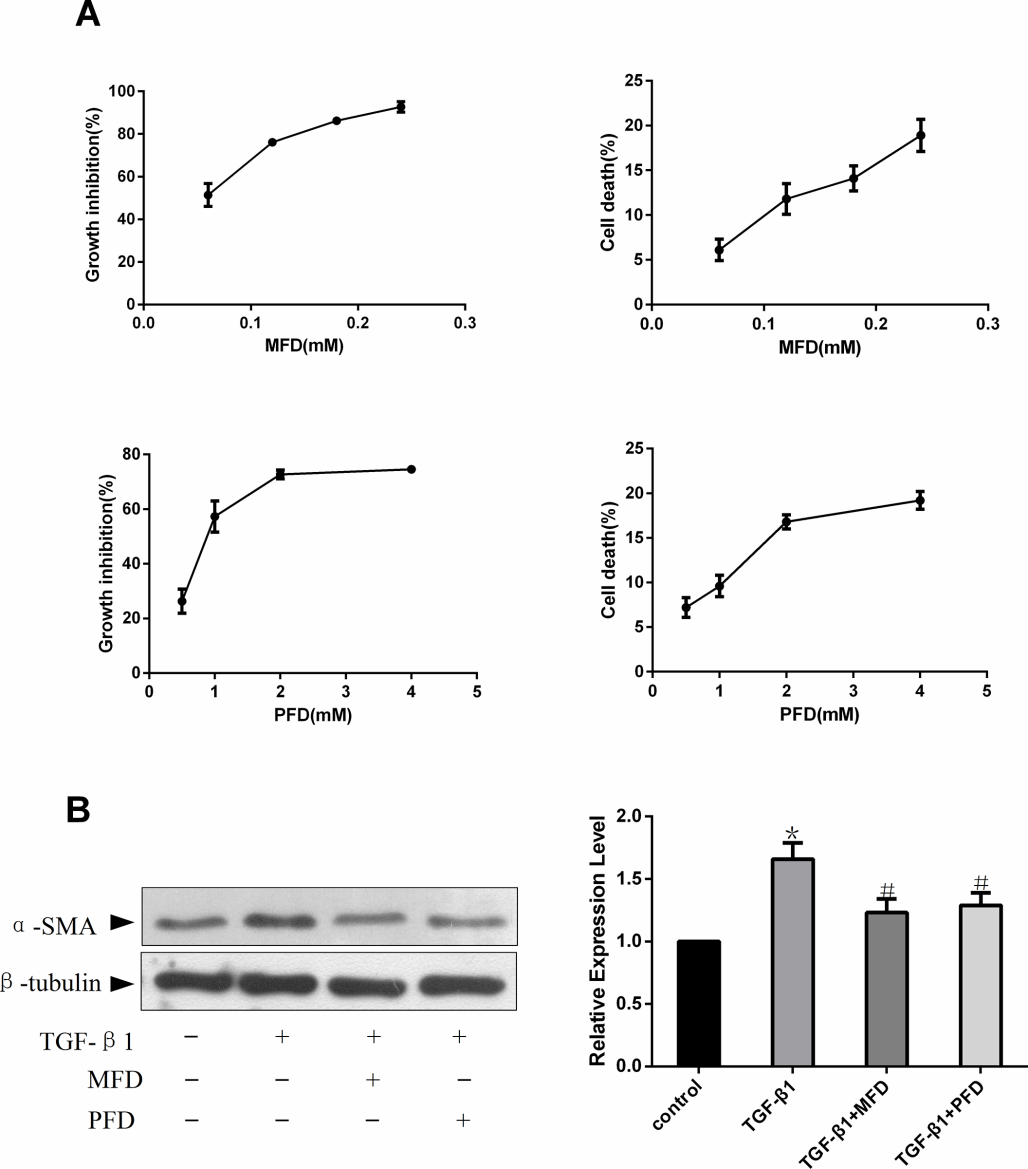
Mefunidone inhibited TGF-β1-induced NRK-49F cell proliferation and α-SMA protein expression. ***A*.** Mefunidone and pirfenidone (PFD) decreased NRK-49F cell viability in a dose-dependent manner measured by bromodeoxyuridine (BrdU) incorporation. The effect of Mefunidone and PDF on cell death of NRK-49F was assessed by LDH Cytotoxicity Assay Kit. ***B*.** Mefunidone reduced α-SMA protein expression in NRK-49F cells shown by Western blot analysis. The column section indicated the relative expression level of each group. The data were presented as means±SD, n = 3. (*p<0.05 compared to the control group; #p<0.05 compared to the TGF-β1 group.)

Based on the finding that Mefunidone could effectively attenuate interstitial fibrosis in UUO animals, we decided to determine the effect of Mefunidone on fibroblast proliferation using NRK-49F cells. As shown in Fig 3A, both Mefunidone and PFD inhibited TGF-β1-induced NRK-49F proliferation in a dose-dependent manner assessed by BrdU incorporation. But the effective dose of Mefunidone was much lower than that of PFD. Meanwhile, we observed that the effect of Mefunidone and PDF on NRK-49F cells were independent of cell toxicity. We further examined the protein expression of the fibroblast activation marker α-SMA. As shown in Fig 3B, Mefunidone and PFD suppressed TGF-β1-induced production of α-SMA in NRK-49F cells.

### Mefunidone attenuated immune cell infiltration in the obstructive kidney after UUO

**Fig 4:**
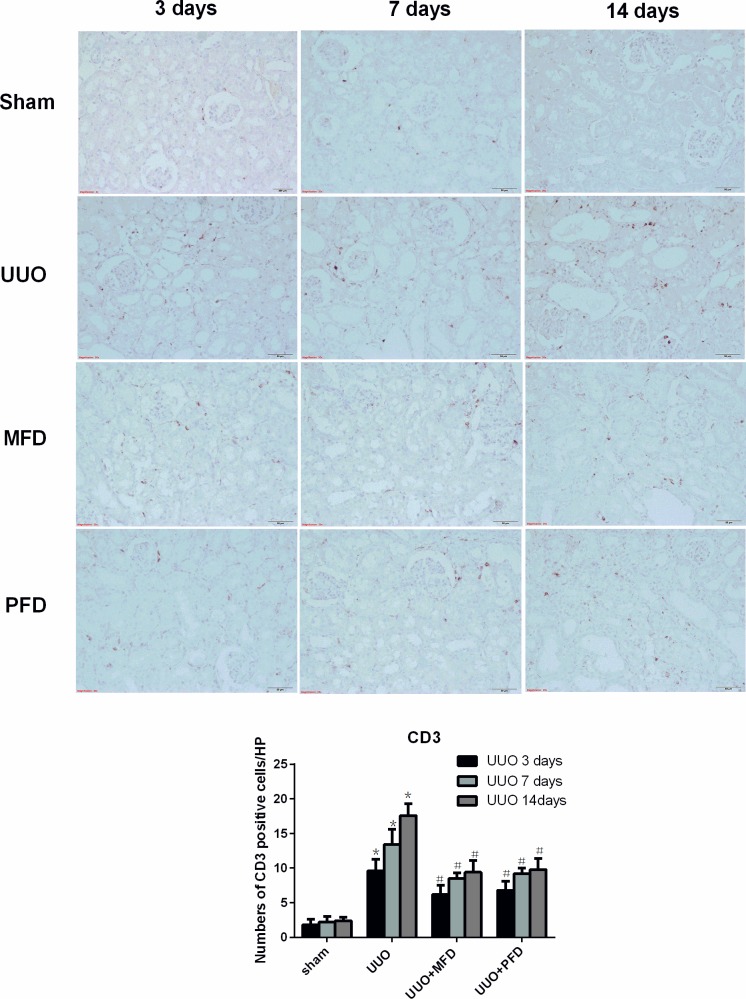
Mefunidone attenuated leukocyte infiltration in the obstructive kidneys after UUO at 3, 7 or 14 days. Leukocytes were stained as CD3 by immunohistochemistry in the obstructive kidneys (×200). The column section indicated the numbers of CD3 positive cells/HP of each group. The data were presented as means±SD, n = 5. (*p<0.05 compared to the sham group; #p<0.05 compared to the UUO group.)

**Fig 5:**
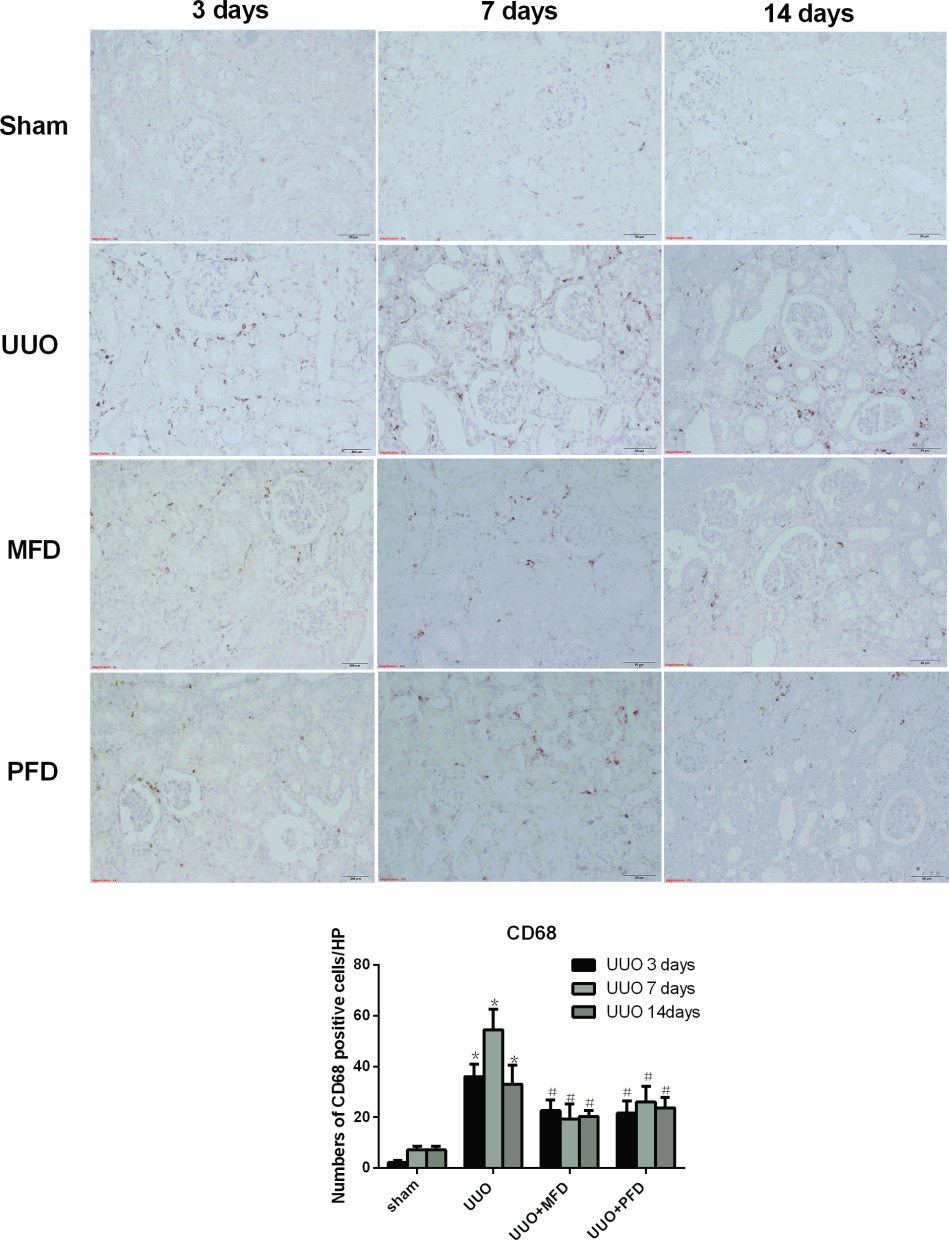
Mefunidone attenuated macrophage infiltration in the obstructive kidneys after UUO at 3, 7 or 14 days. Macrophages were stained as CD68 by immunohistochemistry in the obstructive kidneys (×200). The column section indicated the numbers of CD68 positive cells/HP of each group. The data were presented as means±SD, n = 5. (*p<0.05 compared to the sham group; #p<0.05 compared to the UUO group.)

Tubulointerstitial fibrosis is typically conditioned by the infiltration of inflammatory cells [37] including lymphocytes [38, 39], macrophages [40], dendritic cells [41] and mast cells [42]. Therefore, we next determined whether Mefunidone could inhibit immune cell infiltration to attenuate tubulointerstitial fibrosis. To investigate whether Mefunidone ameliorated lymphocyte and macrophage infiltration, we used immunohistochemical assays for CD3 and CD68 in the obstructive kidneys. CD3- and CD68-positive cells increased in the renal tissues at day 3, 7 and 14 after UUO. We found that Mefunidone and PFD treatment significantly ameliorated the infiltration of lymphocytes and macrophages at day 3, 7 and 14 after UUO (Figs 4 and 5).

### Mefunidone inhibited cytokine release and the phosphorylation of ERK1/2, NF-κB and STAT3 in HK-2 cells and macrophages

**Fig 6:**
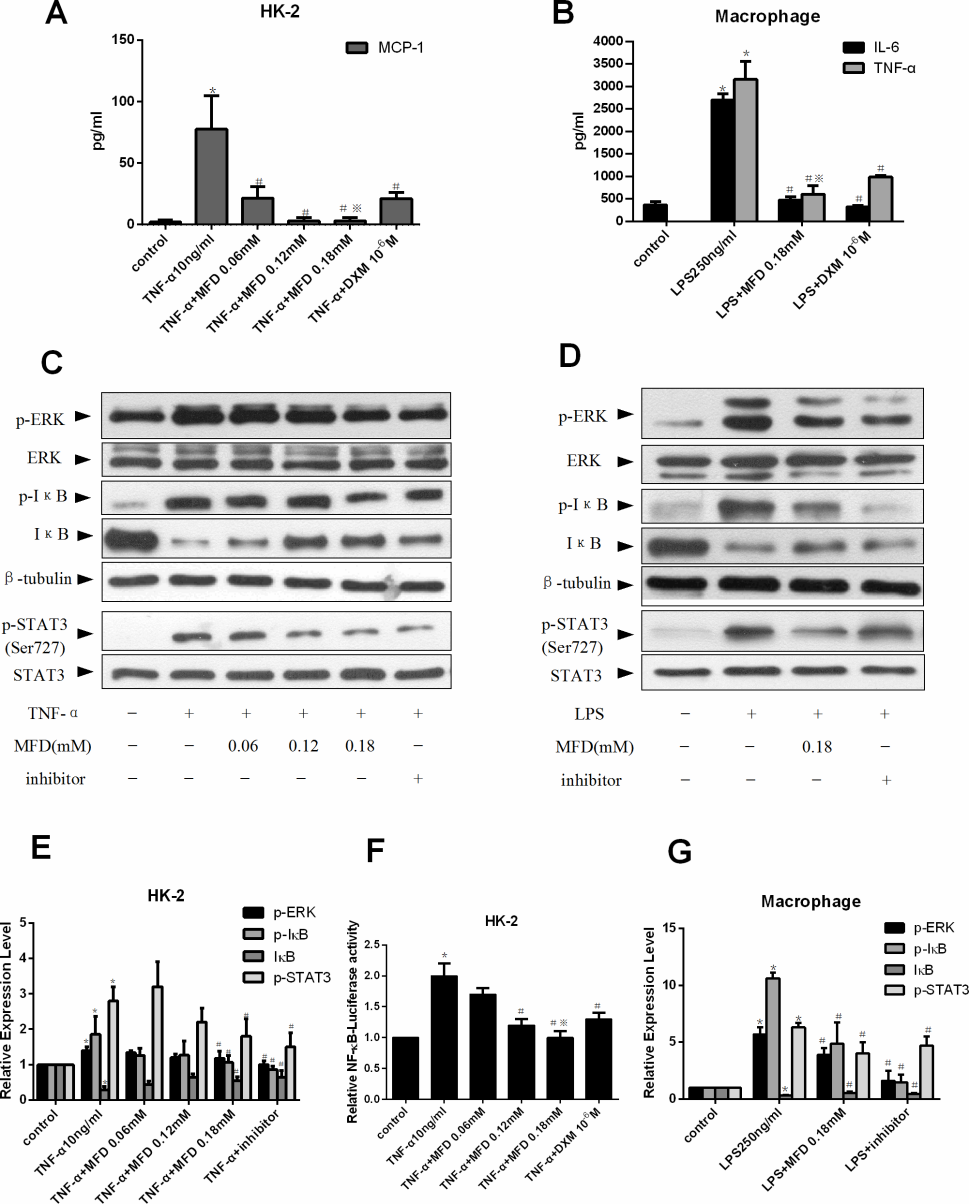
Mefunidone inhibited cytokine release and affected ERK1/2, NF-κB and STAT3 signaling pathways in HK-2 cells and macrophages. ***A*.** Mefunidone inhibited cytokine release in HK-2 cells culture supernatants using enzyme-linked immunosorbent assay (ELISA). ***B*.** Mefunidone inhibited cytokine release in macrophages culture supernatants using ELISA. ***C and E*.** Mefunidone affected the phosphorylation of ERK1/2, NF-κB and STAT3 stimulated with TNF-α in HK-2 cells. The inhibitor was PD98059 (10^−6^μM), BAY (10μM) or AG490 (150μM) respectively. The columnar section was the optical density of the gels tested by Western blot. ***D and G*.** Mefunidone affected the phosphorylation of ERK1/2, NF-κB and STAT3 stimulated with LPS in macrophages. The inhibitor was PD98059 (10^−6^μM), BAY (10μM) or AG490 (150μM) respectively. The columnar section was the optical density of the gels tested by Western blot. ***F*.** Assays of luciferase activity were performed and pNF-κB-luciferase activity was normalized to pRL-SV40 luciferase activity in HK-2 cells. The data were presented as means±SD, n = 3. (*p<0.05 compared to the control group; #p<0.05 compared to the TNF-α or LPS group; ※p<0.05 compared to the DXM group.)

We next determined the mechanism by which Mefunidone inhibited immune cell infiltration during renal interstitial fibrosis. To investigate whether Mefunidone could inhibit the production of inflammatory cytokines directly, HK-2 cells were stimulated with TNF-α in the absence or the presence of different concentrations of Mefunidone or dexamethasone (DXM) for the indicated time. As expected, TNF-α-stimulated MCP-1 production in HK-2 cells culture supernatants using enzyme-linked immunosorbent assay (ELISA) (Fig 6A). Notably, Mefunidone inhibited MCP-1 release in a dose-dependent manner with the maximal effect at the concentrations of 0.18mM (Fig 6A). To further confirm the effect of Mefunidone in inflammation, primary mouse peritoneal macrophages were stimulated with lipopolysaccharide (LPS) for the indicated time. Addition of Mefunidone to cell culture (0.18mM) significantly inhibited IL-6 and TNF-α production. This inhibitory effect was stronger than that of DXM (Fig 6B).

To investigate through which pathway Mefunidone inhibited the production of cytokines and further elucidate the underlying molecular mechanism, HK-2 cells and macrophages were stimulated respectively with TNF-α or LPS in the absence or the presence of different concentrations of Mefunidone for the indicated time. We observed that TNF-α induced the phosphorylation of ERK1/2 in HK-2 cells using western-blot analysis. Addition of Mefunidone (0.18mM) or PD98059, a highly selective inhibitor of MEK1 activation and the MAP kinase cascade, significantly decreased phosphorylation of ERK1/2 (Fig 6C and 6E). Similar observation was made in macrophages. Stimulation of LPS induced the phosphorylation of ERK1/2 in macrophages. Addition of Mefunidone (0.18mM) or PD98059 to macrophage culture significantly decreased phos-ERK1/2 (Fig 6D and 6G).

The NF-κB pathway is another important intracellular signaling pathway related to inflammation. HK-2 cells were stimulated with TNF-α in the absence or the presence of different concentrations of Mefunidone or BAY 11–7082, a selective inhibitor of IκB-α phosphorylation. We observed that addition of Mefunidone (0.18mM) not only decreased the phosphorylation of IκB-α, but also increased the protein expression of IκB-α (Fig 6C and 6E). This phenomenon was confirmed by using macrophages (Fig 6D and 6G). We next determined whether Mefunidone could affect the transcription activity of NF-κB in the presence of TNF-α. We transiently transfected HK-2 cells with a NF-κB reporter plasmid and then stimulated the genetically modified cells with TNF-α in the presence or the absence of Mefunidone or DXM. As shown in Fig 5F, the luciferase assay showed that exposure of HK-2 cells to TNF-α (10ng/ml) resulted in upregulation of NF-κB activity. Addition of Mefunidone (0.12mM and 0.18mM) significantly inhibited the transactivation of NF-κB induced by TNF-α.

Evidence suggests that the JAK-STAT pathway also plays an important role in inflammation. Previous works demonstrated that the JAK2-STAT3 pathway contributes to the pathogenesis of fibrotic renal diseases [43]. Therefore, we next investigated the effect of Mefunidone on JAK2-STAT3. HK-2 cells were stimulated with TNF-α in the absence or the presence of different concentrations of Mefunidone or AG490, a special JAK2 and STAT3 inhibitor. We observed that TNF-α induced the phosphorylation of STAT3 (Ser727) in HK-2 cells using western-blotting. Addition of Mefunidone (0.18mM) or AG490 inhibited phosphorylation of STAT3 (Ser727). Further, we obtained a similar phenomenon in macrophages. (Fig 6C, 6D, 6E and 6G)

## Discussion

To confirm the anti-fibrotic effect of Mefunidone, we investigated the effect of Mefunidone on tubulointerstitial injury score, ECM deposition, collagen expression and fibroblast activation in UUO models. The results in the present study showed that Mefunidone attenuated tubulointerstitial injury in a dose-dependent manner. Notably, the tubulointerstitial injury score of the high-dose (100mg/kg/d) Mefunidone treatment groups was markedly lower than that of the PFD group. In addition, treatment of Mefunidone markedly decreased size of the Sirius red staining positive area and the score of interstitial ECM deposition. Immunostaining and real-time PCR revealed that treatment of Mefunidone inhibited interstitial collagen I and collagen III expression in the kidneys after UUO. We also observed that Mefunidone could inhibit fibroblast activation in the obstructive kidneys, which was proved by detecting the protein expressions of α-SMA and FN. These results suggested that this novel compound might be a new candidate for treating renal fibrosis. Fibroblasts in the renal interstitium are considered the principal source of fibrillar matrix. We presented the second piece of evidence that Mefunidone could inhibit TGF-β1-induced NRK-49F (a rat kidney fibroblast) proliferation in a dose-dependent manner assessed by BrdU incorporation and suppressed the production of α-SMA increased by TGF-β1 in NRK-49F.

The inflammation exists throughout the development of CKD. Inflammatory cell infiltration plays an important role in chronic inflammation and fibrosis in kidney. Some scholars found an infiltration of macrophages in the obstructed kidneys at 4h after UUO and these activated inflammatory cells released inflammatory mediators such as TNF-α, which, in turn, activated fibroblasts and initiated renal fibrosis [20, 44]. Therefore, we examined whether Mefunidone could inhibit the inflammatory response and subsequent renal fibrosis. Our data showed that Mefunidone significantly ameliorated lymphocyte and macrophage infiltration in the obstructive kidney.

Accordingly, Mefunidone significantly inhibited TNF-α-stimulated MCP-1 release in HK-2 cells and LPS-stimulated IL-6 and TNF-α release in macrophages *in vitro*, indicating that Mefunidone could directly inhibit the inflammatory response. Although the pathogenesis of renal fibrosis is not fully understood, accumulating evidence indicate that immune cell infiltration and excessive production of inflammatory cytokines importantly contribute to renal fibrosis [19, 20]. Therefore, Mefunidone treatment attenuated renal interstitial fibrosis, at least in part, through inhibition of the inflammation. The JAK-STAT, ERK1/2, and NF-κB signaling pathways play pivotal roles in the inflammatory responses [23, 24]. The present study showed that Mefunidone could inhibit TNF-α- or LPS-induced phosphorylation of ERK1/2, STAT, and IκB. We also proved that Mefunidone could effectively inhibit the activation of NF-κB. These results provide mechanistic insight that Mefunidone inhibits inflammation via suppressing the activation of ERK1/2, JAK-STAT and NF-κB signaling pathways. As we know, there is a complex crosstalk between these three pathways [45, 46]. Interestingly, we found that TNF-α or LPS could induce the phosphorylation of STAT3 Ser727 but not Tyr705 in HK-2 cells or macrophages. However, further investigation is needed to make it clear for the exact target of the anti-fibrotic and anti-inflammatory effect of Mefunidone, such as whether there are possibly additional anti-fibrotic effects that are independent of the anti-inflammatory effects, whether Mefunidone affects the STAT3 Ser727 phosphorylation by affecting the ERK1/2 and/or NF-κB phosphorylation.

In conclusion, Mefunidone significantly attenuates renal interstitial fibrosis in a rat model of UUO. The anti-fibrotic effects of Mefunidone may be related to the regulation of inflammatory cell infiltration and release of inflammatory factors achieved by affecting ERK1/2, STAT3, and NF-κB signaling pathways. Mefunidone may be a new candidate for the treatment of renal fibrosis.

## Supporting Information

S1 Fig The effect of Mefunidone on serum creatinine and urea nitrogen in the rats after UUO at 14 days. (TIF)

S2 Fig The effect of Mefunidone on ERK1/2 and NF-κB signaling pathways in HK-2 cells compared to PFD. (TIF)
